# Fast and slow gene expression changes in blood following acute social stress

**DOI:** 10.1016/j.bbih.2026.101365

**Published:** 2026-09-17

**Authors:** Hampus Grönvall, Sylvia Abdelhalim, Fara Tabrizi, Sini Ezer, Gamze Yazgeldi Gunaydin, Erik Arner, Jörgen Rosén, Shintaro Katayama, Juha Kere, Fredrik Åhs, Lea Mikkola

**Affiliations:** a Department of Education, Psychology and Social Work, Mid Sweden University, Östersund, Sweden; b Department of Clinical Neuroscience, Karolinska Institutet, Stockholm, Sweden; c Folkhälsan Research Centre, Helsinki, Finland; d Stem Cells and Metabolism Research Program, University of Helsinki, Helsinki, Finland; e Department of Medicine, Huddinge, Karolinska Institutet, Stockholm, Sweden; f RIKEN, Yokohama, Japan; g Turku Bioscience Centre, University of Turku, Turku, Finland; h InFLAMES Research Flagship, University of Turku, Turku, Finland

## Abstract

Social stress is a risk factor for psychiatric disorders and also influences immune function. While it is known that acute social stress impacts the number of immune cells in circulation, the temporal dynamics of stress induced immune-related transcriptional changes in human blood remain unclear. To investigate changes in gene expression, we exposed 27 adults to the Trier Social Stress Test (TSST), and collected blood at baseline, as well as 5, 30, 60 and 90 min after stress. Whole-blood gene expression was profiled using a 5’ targeted RNA-sequencing method (STRT). Differential expression was analyzed using linear and cubic models. We observed a total of 54 differentially expressed genes following stress. Fast responses, with a transient peak immediately following stress, were enriched for cytotoxic T-cell, NK-cell and dendritic cell functions (e.g., *GZMB, GNLY, CCL4* and *GZMA*) and paralleled lymphocyte count changes. In contrast, gradual, linear responses without any evident peak were enriched for neutrophil related genes (e.g., *FPR2, PLAUR, CXCR2, AQP9*, and *QPCT*) and did not mirror neutrophil counts, indicating cell intrinsic transcriptional changes. From pathway and transcription factor enrichment analyses the IL-12 family mediated signaling is inferred as a mechanism linking stress to immune gene regulation. Our results showed both fast and slower changes in gene expression in different immune cell populations following acute psychosocial stress. The involvement of the IL-12-STAT4 axis and genes such as *PLAUR* and *FPR2* suggests molecular mechanisms through which stress-related immune activation may contribute to vulnerability for anxiety and depressive disorders.

## Highlights

• Acute stress induces distinct fast and slow immune gene responses.
• 54 differentially expressed genes identified using linear and cubic temporal models.
• Fast responses reflect cytotoxic T/NK activity and mirror lymphocyte shifts.
• Slow responses involve neutrophil genes, independent of cell counts.
• IL-12-STAT4 signalling implicated as regulatory mechanism.

## 1 Introduction

Stress related psychiatric disorders impose a heavy burden on both individuals and society. An understand of the biological pathways linking stress to risk for psychiatric disorders is needed for finding new ways to inform prevention and intervention efforts. Chronic stress can influence the immune system, potentially increasing risk for depression and anxiety via inflammatory mechanisms (Slavich and Irwin, 2014). Higher circulating levels of immune markers such as IL-6 and C-reactive protein have been associated with depression (Milaneschi et al., 2021; Pitharouli et al., 2021; Roohi et al., 2021). It has therefore been hypothesized that acute stress triggers gene expression changes in immune cells, and that repeated or prolonged activation of these immune pathways may contribute to the development of depression or anxiety after chronic stress exposure.

Prior studies that have used targeted approaches to study gene expression have shown that acute psychosocial stress can increase the expression of immune-related proteins such as the transcription factor NF-κB and cytokines IL-1β and IL-10 (Brydon et al., 2005; Kuebler et al., 2015). While these findings point to stress induced activation of inflammatory signaling, unbiased whole-transcriptome studies are needed to discover novel stress responsive molecular pathways. To our knowledge, four genome wide transcriptomic studies of acute stress have been reported (Jurkiewicz et al., 2021; Logan et al., 2022; Nater et al., 2009; Schwaiger et al., 2016). These studied all employed the Trier Social Stress Test (TSST) (Kirschbaum et al., 1993), but differed in sample characteristics (e.g., healthy volunteers vs. individuals with trauma history) and post-stress sampling times, ranging from immediately after stress to 3 h later. Furthermore, the specific genes showing the largest changes have been inconsistent between studies.

A major limitation of previous studies is the lack of concurrent immune cell count measurements alongside gene expression profiling. Acute stress is well known to cause rapid increases in circulating neutrophils, lymphocytes and monocytes, with neutrophils remaining elevated for an h or more following stress (Chan et al., 2023; Obeagu, 2025; Poller et al., 2022). Such shifts in blood cell composition can confound bulk gene expression results. For example, when MacCormack et al. (2021) adjusted for estimated cell-type proportion, many stress related gene expression differences became non-significant, suggesting that those signals were driven by changes in cell counts rather than gene regulation. Another gap in prior research is limited temporal resolution. Most studies collected at most one or two post stress samples, making it difficult to distinguish early gene responses from late responses.

To this end, we characterized whole genome changes in gene expression following stress by sequencing RNA isolated from whole blood at baseline, and at 5, 30, 60 and 90 min after stress. To compare changes in gene expression with changes in blood composition, we also measured immune cell counts at the same timepoints. This design allowed us to investigate which genes changed in activity, both early and late after stress.

## 2 Subjects and methods

### 2.1 Participants

Participants were recruited from the Swedish Twin Registry (Zagai et al., 2019) as part of the larger TwinStress research project. The study sample consisted of 27 participants (17 female, 10 male), between 32 and 58 years of age (M = 42, SD = 7.3), comprising eight monozygotic (MZ) and one dizygotic (DZ) same sex pairs, along nine singletons. Exclusion criteria for participation were currently on-going pregnancy or severe mental illness. Participants underwent screening through self-report and a structured psychiatric interview. Additionally, participants were instructed to not consume caffeine or nicotine on the day of participation, and to avoid intensive exercise for at least 48 h prior. Full sample characteristics are available in the supplementary materials. All participants signed informed consent, and the study was approved by the Swedish Ethical Review Authority (Dnr. 2020-00885).

### 2.2 Power calculation

This study uses the first data freeze from the larger TwinStress project. No formal a priori power calculation was performed for the FDR-controlled transcriptomic analyses, and thus this study should be considered exploratory. A power calculation using G*Power showed that for a standard repeated measures within-factor model, a sample size of *N* = 26 was needed to detect a medium effect size (*f* = .25) with a power of .90.

### 2.3 Procedure

To account for diurnal variation in cortisol levels, all sessions were conducted at approximately the same time of day, starting between 11:00 to 12:00. Upon arrival, a catheter was inserted in the non-dominant arm for blood sampling and participants were given a brief rest period before undergoing the Trier Social Stress Test (TSST) (Kirschbaum et al., 1993). The TSST followed the classic procedure (Allen et al., 2017) consisting of a simulated job interview in front of a two-person panel. Participants were given 3 min to mentally prepare a 5 min speech about themselves. During the speech, participants were told that there was still time left and to continue speaking, if they were to hesitate or be silent for 15 s consecutively. This was then followed by an unprepared 5-min mental arithmetic task (counting backward from 2322 in steps of 13 as quickly and correctly as possible). Were they to make a mistake, they were instructed to start over from 2322. To enhance social-evaluative threat, the panel maintained neutral expressions and gave minimal feedback during the TSST while taking mock notes. Participants were also informed that their performance would be recorded through a visibly present video camera for later evaluation. No video recordings were saved. To asses subjective stress levels, the State-Trait Anxiety Inventory (STAI-S) (Spielberger et al., 1983) was deployed before the task, after the 3 min preparation phase, upon completion of the arithmetic task, and at the end of the recovery period (90 min post stress).

### 2.4 Blood sampling

Venous blood was collected at five timepoints: approximately 30 min prior to the TSST (baseline), and then at 5, 30, 60 and 90 min following the completion of the TSST. For the first three timepoints, plasma was isolated and analyzed for cortisol levels through electrochemiluminescence immunoassay (ECLIA) using a Roche Cobas e801 analyzer (Roche Diagnostics, Mannheim, Germany). Whole blood and plasma samples were stored refrigerated at 4 °C for a maximum of 24 h before being analyzed. Complete blood counts were determined using a Sysmex XN-series hematology analyzer (Sysmex Corporation, Kobe, Japan). These analyses were performed by the Karolinska University Laboratory at Karolinska Institutet, Stockholm.

### 2.5 Statistical analyses of perceived stress, cortisol levels and cell counts

To investigate changes across time in perceived stress, cortisol levels and blood cell counts, we fitted linear mixed-effects models using the lmerTest version 3.1.3 (Kuznetsova et al., 2017) in R version 4.5.1 (R Core Team, 2025). Each model included timepoint as a fixed factor and participant ID as a random intercept to account for within-person dependencies. Tests of fixed effects were based on Type III F-tests with Satterthwaite-approximated degrees of freedom. Post-hoc contrasts between consecutive timepoints were estimated using emmeans version 2.0.1 (Lenth, 2017), with *p*-values adjusted for multiple comparisons via the Benjamini-Hochberg procedure within each outcome and effect sizes (*d*) from the model residual standard deviation and its estimated degrees of freedom. Visualisations were created with ggplot2 version 4.0.1 (Wickham, 2016).

### 2.6 Preprocessing, cleaning and quality assessment of STRT sequencing libraries

Whole-blood gene expression was profiled using single-cell tagged reverse transcription sequencing (STRT-Seq). Raw sequencing reads were processed with the STRT-N pipeline (Boskovic et al., 2023). Sequencing reads were mapped to the reference human genome (hg38) and annotated using the GENCODE v43 basic annotation set (Frankish et al., 2021) via the UCSC Genome Browser (Perez et al., 2024). We computed standard library-specific quality control (QC) metrics for each of the four multiplexed sequencing libraries (each containing 48 samples), including total mapped reads, spike-in reads, and 5′-end alignment rates, overall mapping ratio, ratio of mapped reads to spike-ins, and 5′-end coding read percentage. Based on these metrics outlier samples, non-template controls (NTCs), and technical duplicates were excluded from both libraries (see Supplementary Figure S1). Following quality control, 42, 34, 37 and 40 samples were retained for downstream analyses from each of the four libraries respectively.

Gene-level count matrices were generated after read alignment. Genes with zero counts in > 5 samples were filtered out to eliminate very low-expressed transcripts. Initially, 19 761 genes were detected, and after filtering 12 700 genes remained. Because our samples were sequenced in multiple library batches, we applied a method to correct for potential library-specific biases. Specifically, we used the Negative Binomial Generalized Linear Model – Library Bias Correction (NBGLM-LBC) package (Katayama et al., 2019) in R version 4.2.2 (R Core Team, 2025) to adjust gene counts between libraries. Read depth information was extracted from BAM files using samtools (Danecek et al., 2021). We next performed spike-in-based variability analysis to identify genes with significant fluctuations across samples (Krjutškov et al., 2016). A total of 1171 genes showed high variability (Benjamini-Hochberg FDR corrected *p* < .05 in the spike-in variability test) and were labelled as fluctuating genes to be used in downstream analyses. Principal component analysis (PCA) was carried out on both raw and bias corrected count data using the Seurat package version 5.0.1 (Hao et al., 2024) to verify that library batch effects were effectively mitigated after correction (see Supplementary Figure S2). Following QC and batch correction, gene counts were spike-in normalized with log-transformation.

### 2.7 Differential gene expression analysis

Differential gene expression (DGE) analysis was carried out on the filtered and bias-corrected dataset of fluctuating genes using the R packages edgeR version 4.2.2 (Chen et al., 2025) and limma version 3.60.6 (Ritchie et al., 2015). We applied trimmed mean of M-values (TMM) normalization to account for library size differences. To capture potential non-linear trends in gene expression, we fit two separate regression models: a linear model and cubic polynomial model. In the linear model, expression was modeled as a function of time (in min) relative to TSST completion, treated as a continuous variable. In the cubic model, time was modeled with a third-degree (cubic) polynomial to allow for transient expression changes that peak and subside during the study window. Both models were applied to log_2_ counts-per-million values. Because our sample included for twin pairs, we accounted for within-pair non-independence using limma's duplicate correlation function. From the resulting lmFit models, gene expression statistics were computed using limma's eBayes function. Genes with Benjamini-Hochberg FDR corrected *p*-value < .05 in either model were deemed significantly differentially expressed.

### 2.8 Gene set enrichment, transcription factor enrichment, and protein-protein interaction analyses

To investigate enriched terms and pathways, we performed gene set enrichment analysis (GSEA), transcription factor enrichment analysis (TFEA), and protein-protein interaction analysis on the combined set of significant DE genes from both linear and cubic models (removing duplicates).

GSEA was performed using the g:Profiler web tool (Kolberg et al., 2023) version e113_eg59_p19_6be52918 with g:OSt and default parameters. The results were then visualized using the multienrichjam version 0.0.86.900 (Farris et al., 2019), ggplot2 version 4.0.1 (Wickham, 2016) and enrichplot version 1.28.4 (enrichplot, 2025) packages alongside base R plotting functions. For TFEA, the ChEA3 (Keenan et al., 2019) transcription factor enrichment tool was used. The relationship between TFs and target genes from ChEA3 were then identified by clustering and visualization using Clustergrammer (Fernandez et al., 2017). Finally, STRING version 12.0 (Szklarczyk et al., 2023) was used to investigate protein-protein interaction predictions. A full interaction network was generated using a medium confidence score cutoff (interaction score ≥ 400).

## 3 Results

### 3.1 Perceived stress

Perceived stress levels varied across the protocol (*F*_*3,71*_ = *17.99, p* < .001, $\eta_{p}^{2}$ = .43, Fig. 1), with an increase from baseline (M = 35.60, SD = 10.95) to after the preparation phase of the TSST (M = 42.56, SD = 13.13, *t*_*71*_ = 3.18, *p* < .05, *d* = .90). It then remained elevated after completion of the TSST (M = 44.58, SD = 12.75, *t*_*71*_ = 1.02, *p = *.31, *d* = .29) followed by a decrease at the end of the recovery period (M = 30.32, SD = 7.96, *t*_*71*_ = −6.52, *p* < .05, *d* = −1.77).

**Fig. 1:**
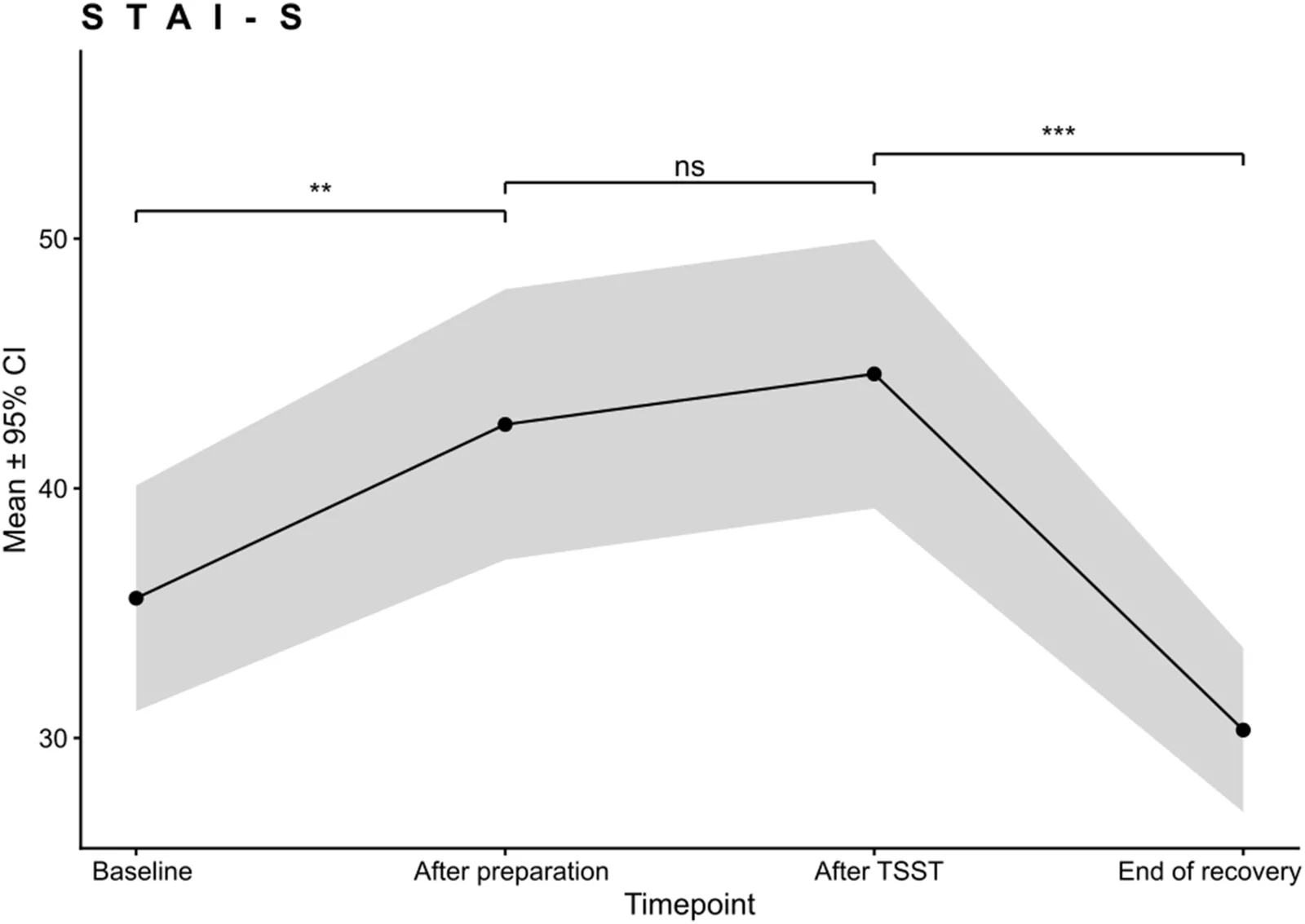
Subjective Ratings of Perceived Stress. Line represents mean STAI-S score with shaded area representing 95 % confidence intervals at each timepoint. Significance levels for consecutive contrasts between timepoints are labelled: **p < .01, ***p < .001, ns = non-significant.

### 3.2 Blood cortisol levels and cell counts

Cortisol varied significantly over time (*F*_2,49_ = 20.71, *p* < .001, $\eta_{p}^{2}$ = .46, Fig. 2A). Consistent with the expected physiological stress response, there was an increase from baseline (M = 262.42, SD = 84.96) to 5 min after stress (M = 378.39, SD = 129.87, *t*_*49*_ = 6.43, *p* < .05, *d* = 1.78), followed by a decrease at 30 min after stress (M = 323.76, SD = 124.45, *t*_*49*_ = −2.91, *p* < .05, *d* = −.82).

**Fig. 2:**
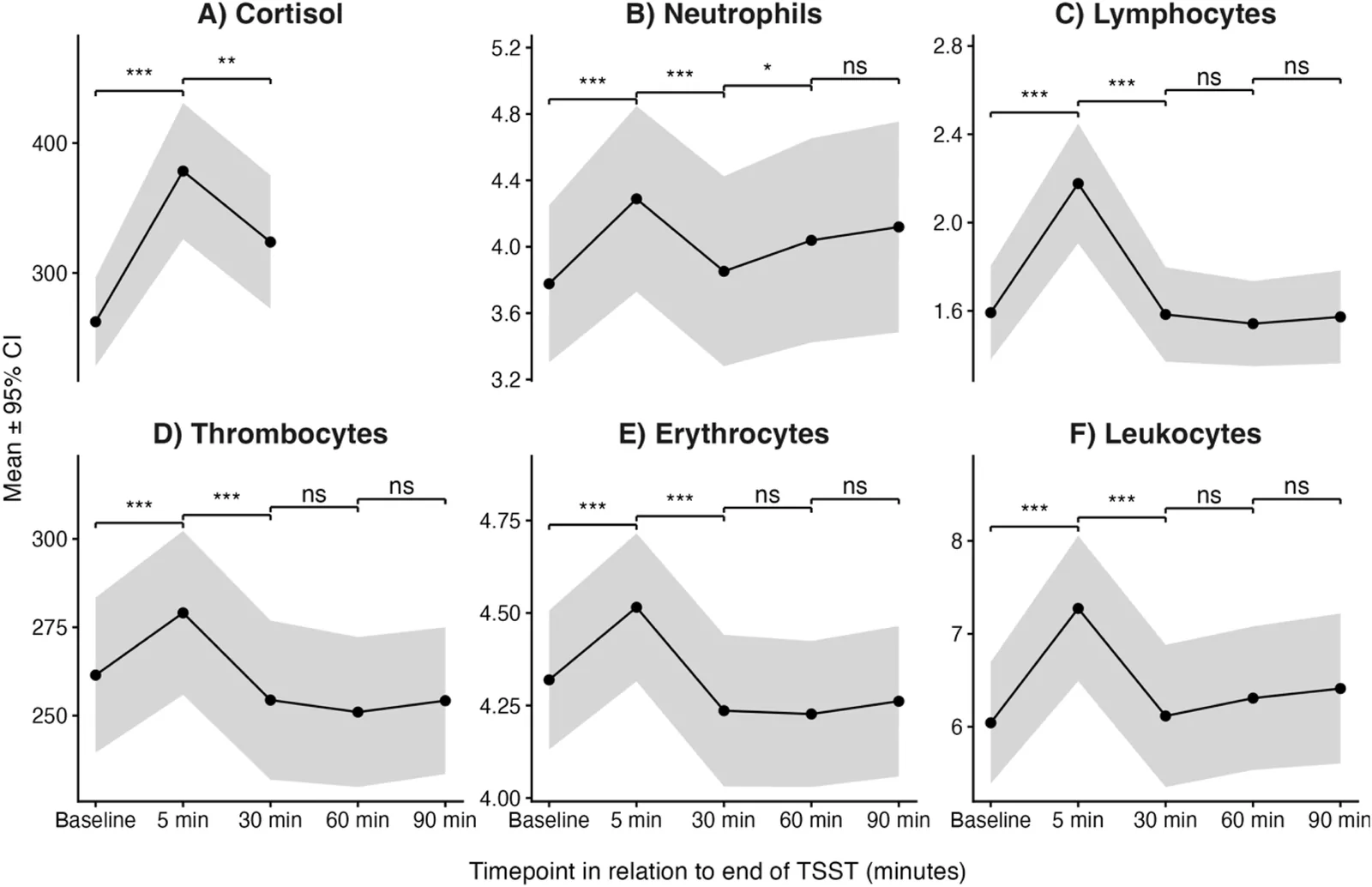
Cortisol Levels and Immune Cell Counts. Line represents mean blood cortisol (nmol/L) and immune cell counts (10^9^/L) with shaded area representing 95 % confidence intervals at each timepoint. Significance levels for consecutive contrasts between timepoints are labelled: *p < .05, **p < .01, ***p < .001, *ns* = non-significant.

As shown in Fig. 2B–E, all cell counts increased immediately after stress, before returning toward baseline. Neutrophil counts then increased again from 30 to 60 min, while other measures remained unchanged. Observed means, fixed effect estimates and contrasts across all timepoints available in Supplementary Table S1-3.

### 3.3 Fast and slow gene expression changes after acute stress

We identified a total of 54 genes that were differentially expressed (DE) over time following the TSST (FDR adjusted *p-*value < .05 in at least one model; see Table 1 for the top differentially expressed genes and Supplementary Tables S4–5 for the full results).

**Table 1:** **Top Differently Expressed Genes.** The five highest ranking genes by Log_2_ FC in either direction uniquely detected by the linear or cubic models, as well as the four genes that were found in both models. For linear models, Log_2_ FC represents change per min, while for polynomial models Log_2_FC represents spline components and do not have a straightforward biological interpretation. For this reason, Log_2_FC and p-values from the linear models are reported for the genes found in both models. Fast change genes indicate genes with a transient spike at 5 min post stress, detected by cubic model. Slow change genes indicate genes showing gradual change over 90 min without any obvious spike, detected by the linear model. Single Cell Type Cluster labels from The Human Protein Atlas (Karlsson et al., 2021). Reported p-values are Benjamini-Hochberg FDR adjusted.

Gene	Single Cell Type Cluster	Change	Model	Log_2_ FC	*p*
GNLY	NK-cells & T-cells - Adaptive immunity: Cytotoxicity	Fast	Both	−.005	.014
GZMB	Plasmacytoid dendritic cells - Viral sensing and interferon response	Fast	Both	−.004	.029
CCL4	NK-cells & T-cells - Adaptive immunity: Cytotoxicity	Fast	Both	−.004	.026
GZMA	NK-cells & T-cells - Adaptive immunity: Cytotoxicity	Fast	Both	−.003	.014
PILRB	Non-specific - Mixed function	Fast	Cubic	2.178	.029
TRDJ1	Not detected – No cluster assigned	Fast	Cubic	2.383	.010
NKG7	NK-cells & T-cells - Adaptive immunity: Cytotoxicity	Fast	Cubic	2.490	< .001
FCRL6	NK-cells & T-cells - Adaptive immunity: Cytotoxicity	Fast	Cubic	2.801	< .001
FGFBP2	NK-cells & T-cells - Adaptive immunity: Cytotoxicity	Fast	Cubic	2.929	.002
SULT1A1	Neurons - Synaptic function & neurogenesis	Slow	Linear	.004	.044
CXCR2	Neutrophils - Innate immunity signal integration	Slow	Linear	.004	.017
QPCT	Neutrophils - Phagocytosis & degranulation	Slow	Linear	.004	.014
AQP9	Neutrophils - Chemotaxis & Apoptosis	Slow	Linear	.004	.014
FPR2	Neutrophils - Chemotaxis & Apoptosis	Slow	Linear	.004	.014

In the linear model, we found a total of 44 genes characterized by gradual changes over time following stress (Fig. 3, upper panels), which we classify as “slow responding”. Of these, 23 were upregulated and 21 were downregulated, with gene expression changes between the first and last measure varying between .60 and −.48 counts per million per min (log_2_FC * 120 min). Using the cubic model, we found 14 genes characterized by non-linear trajectories, showing a significant peak immediately following stress before stabilizing at baseline levels at subsequent measurements (Fig. 3, lower panels). Genes following this pattern of expression were classified as “fast responding”. Four genes were found to be significant in both models. These genes initially peaked after stress, but declined below baseline at later measurements, leading to a negative linear slope being detected despite the overall non-linear trajectory.

**Fig. 3:**
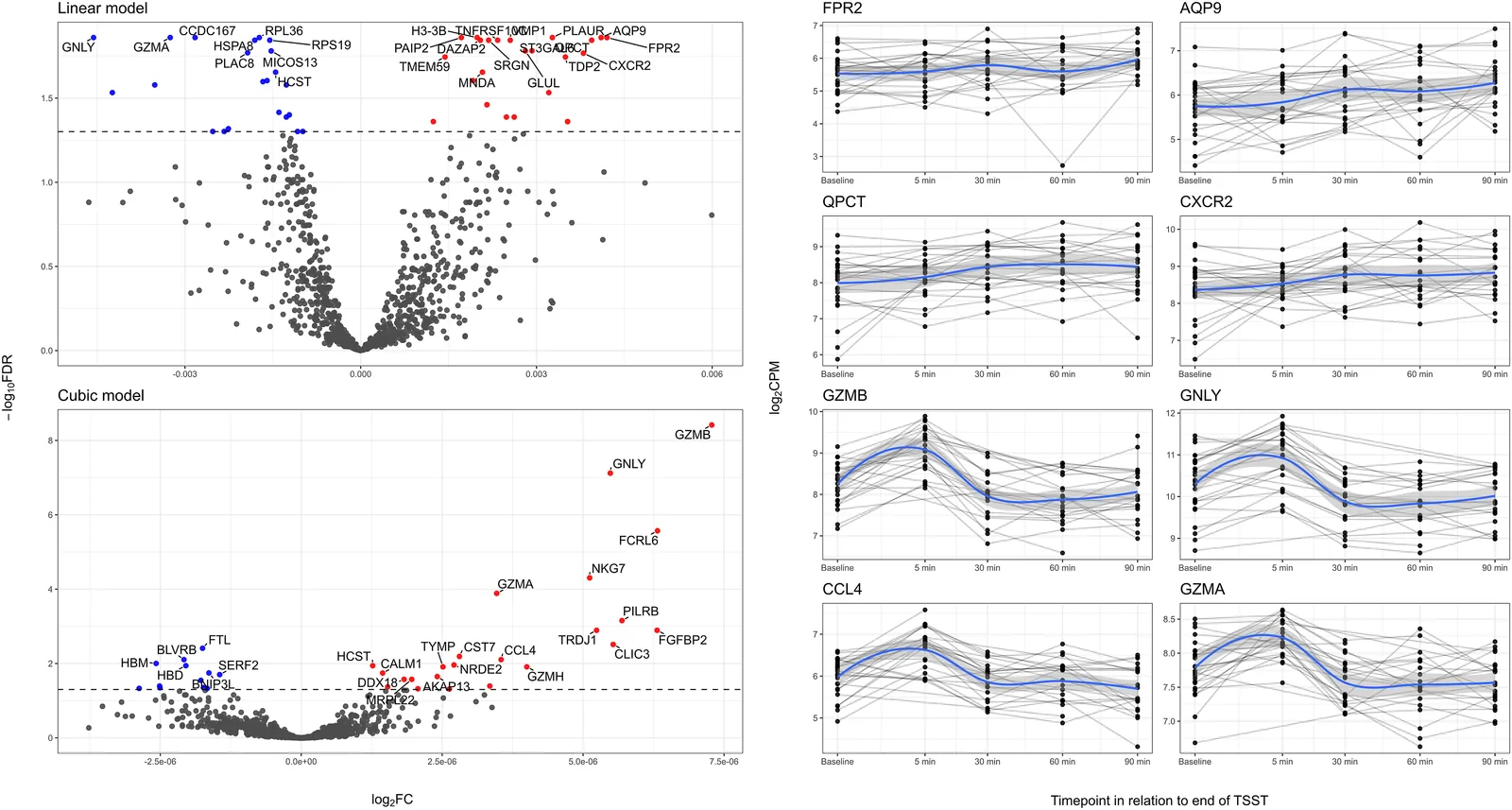
Time Dependent Gene Expression. Left panels: volcano plots of limma-voom differential expression models testing change over time. The upper plot shows the linear time model, and the lower shows the raw cubic time model. Points represent genes. The y-axis is -log_10_ FDR-corrected *p*-values, and the x-axis is log_2_ fold change. The dashed horizontal line indicates the false discovery rate threshold (FDR adjusted *p* < .05). Highlighted and labelled genes denote the most significant hits. Right panels: expression trajectories for the top four genes from each model (linear: *FPR2*, *AQP9*, *QPCT*, *CXCR2*; cubic: *GZMB*, *GNLY*, *CCL4*, *GZMA*). Black points show voom-normalized expression (log2 transformed counts per million) at each sampling time with gray connecting repeated measures within individuals. The blue curve indicates loess-smoothed trend. (For interpretation of the references to color in this figure legend, the reader is referred to the Web version of this article.)

Overall, fast responding genes were associated with cytotoxic T-cell and NK-cell activity, and their gene expression trajectories mirrored that of lymphocyte cell counts (compare Fig. 3 lower right panels with Fig. 2C). In contrasts, the slow responding genes were associated with neutrophil activity, but their trajectories did not follow those of neutrophil cell counts (compare Fig. 3 upper right panels with Fig. 2B).

### 3.4 Pathway, transcription factor and interaction analyses

Gene set enrichment analysis (GSEA) highlighted 20 enriched driver terms related to granzyme-mediated cytotoxicity, cytolytic granules and vesicles, and neutrophil degranulation (Fig. 4, full statistics available in Supplementary Table S6). Among the top ranked TFs identified by TFEA were multiple TFs related to IL-12 family signaling (e.g. *STAT4*, *TBX21*, *STAT5B, HLX* and *EOMES*. See Supplementary Table S7 for complete list). Clustering of TFs and target genes separated two distinct clusters (Fig. 5): one cytotoxic T/NK-cell related cluster (including e.g., the genes *GZMA, GZMB, PLAUR, CCL4* and the TFs *STAT4, EOMES, PRDM1*) and one myeloid/neutrophil related cluster (including e.g., the genes *CXCR2, FPR2, AQP9, HRH2* and the TFs *STAT5B*, *NFIL3* and *ARNTL*). Protein-protein interaction analysis using STRING revealed significant enrichment above random expectation, indicating functional relatedness among the differently expressed genes (Fig. 6). Two separate interaction networks and enrichment were identified related to processes such as regulation of extrathymic T-cell differentiation and NK-cell mediated immunity, cytolytic granules, and cytotoxic T-cell pyroptotic processes.

**Fig. 4:**
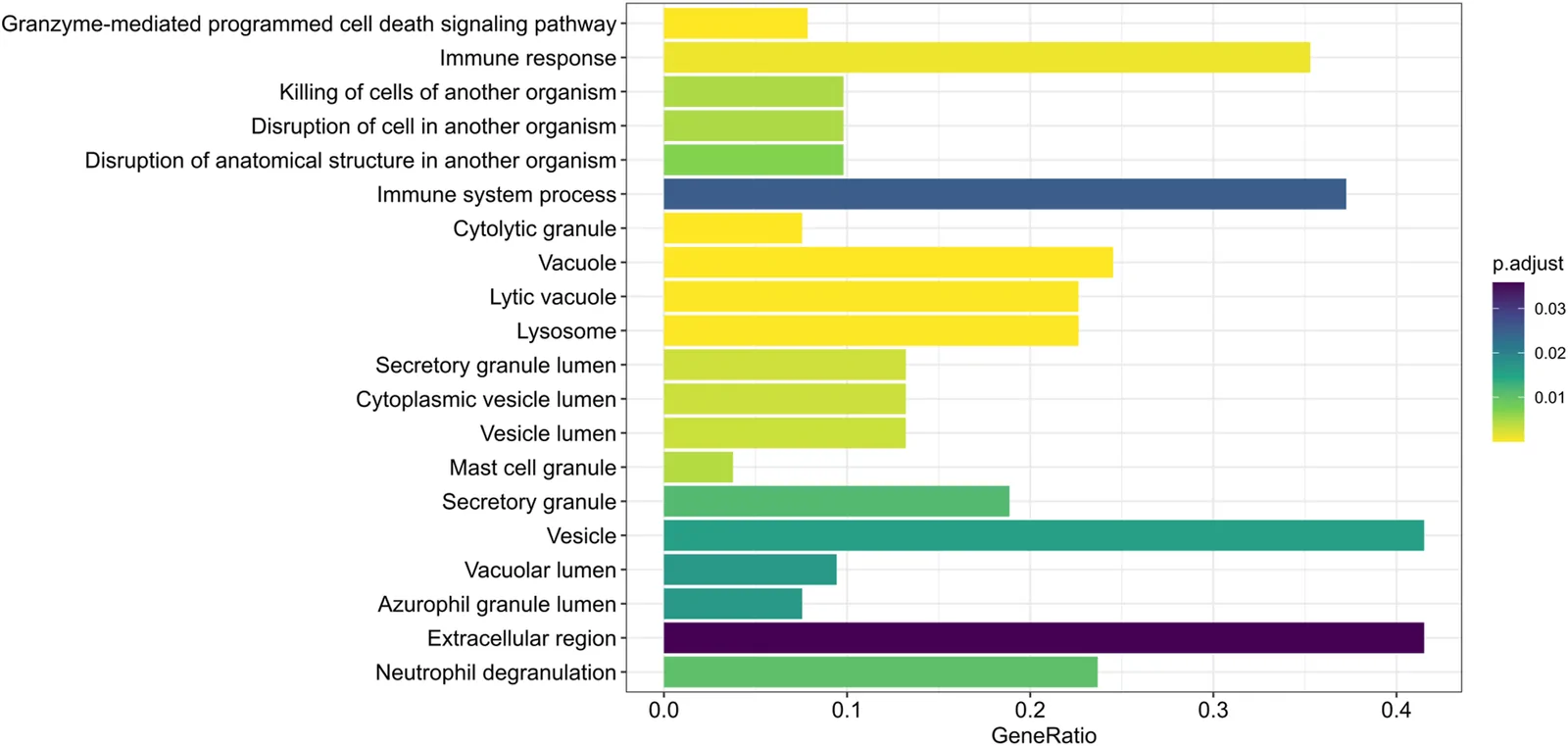
Enriched Terms and Pathways. Bar size on the x-axis indicates gene ratio (intersection size/query size). Color scale refers to significance levels of the gene set enrichment analysis results. (For interpretation of the references to color in this figure legend, the reader is referred to the Web version of this article.)

**Fig. 5:**
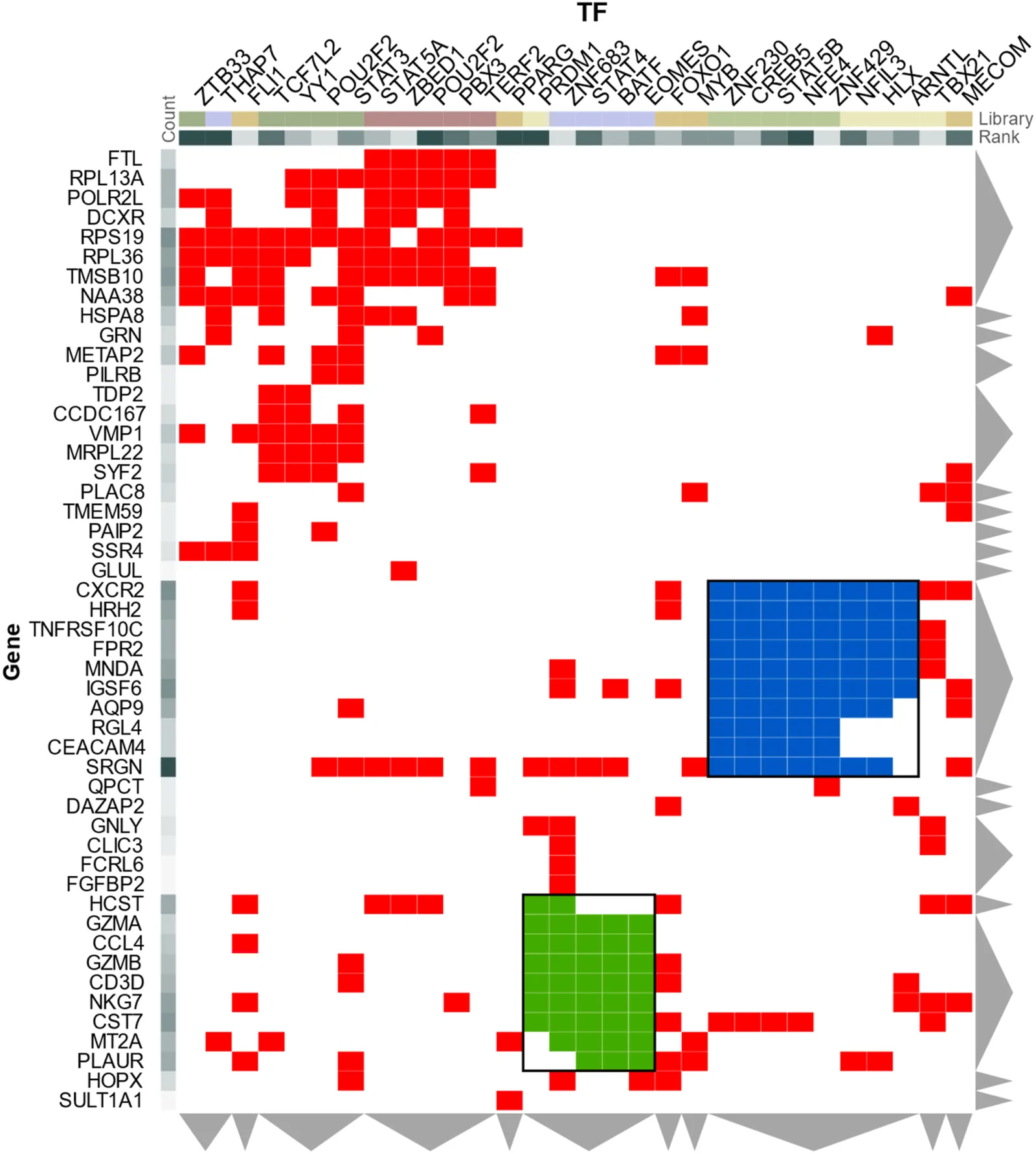
Transcription Factor and Target Gene Clustering. Differently expressed genes are shown on the x-axis and TFs on the y-axis. The cytotoxic T-/NK-cell related cluster has been highlighted blue, and the myeloid/neutrophil related cluster has been highlighted green. (For interpretation of the references to color in this figure legend, the reader is referred to the Web version of this article.)

**Fig. 6:**
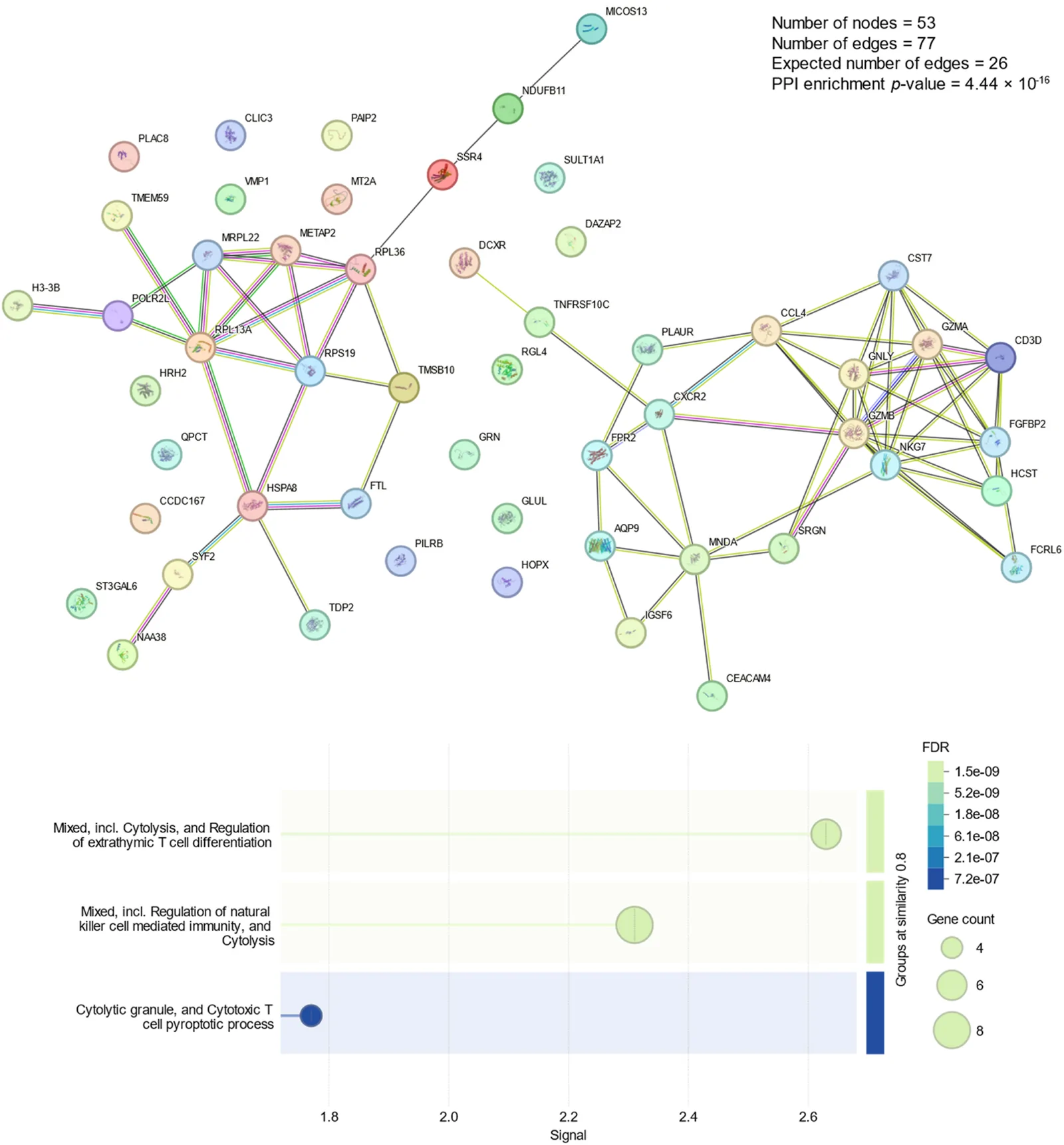
Protein-Protein Interactions. Upper: protein-protein interaction network constructed using STRING, showing known and predicted protein-protein interactions among nodes. Edges represent functional association. Lower panel: bubble plot summarizing gene ontology enrichment for STRING-defined local network clusters. Signal strength is shown on the x-axis, bubble size indicates gene count per term, and color corresponds to FDR adjusted *p*-values. (For interpretation of the references to color in this figure legend, the reader is referred to the Web version of this article.)

## 4 Discussion

This study investigated rapid and delayed changes in gene expression following acute psychosocial stress in humans. We found that fast changes, with a distinct peak following stress, were related to cellular functions of NK-cells and T-cells, whereas slow and linear changes were related to neutrophil activity, consistent with recent mechanistic findings from mouse studies (Poller et al., 2022).

### 4.1 Fast gene expression responses to stress

The rapid changes in gene expression, with a distinct peak immediately after stress, were mainly associated with NK and T-cells, and paralleled the observed cell counts of lymphocytes (of which NK- and T-cells are subsets of). This suggests that, at least in part, the increased expression of these genes may be best explained by concurrent abundance of circulating lymphocytes. For example, we observed increases in the gene expression of granzymes (*GZMA*, *GZMB*), which are key components of granule-mediated cytotoxicity and expressed by multiple immune cells in peripheral blood, including cytotoxic T-cells, NK-cells, and dendritic cells (Karlsson et al., 2021; Voskoboinik et al., 2015). Granzymes are stored in cytolytic granules and can be released during immune activation (Voskoboinik et al., 2015; Lieberman, 2003). Consistent with this, we observed similar transient transcriptional patterns in *NKG7* and *GNLY*, which are both characteristic of cytotoxic lymphocytes and cytotoxic granule biology (Voskoboinik et al., 2015; Ng et al., 2020). Thus, an increase in circulating cytotoxic lymphocytes could contribute to the observed transcriptional changes, even in the absence of strong cell-intrinsic upregulation.

The present findings overlap with earlier TSST transcriptomic studies more clearly at the pathway than at the individual-gene level. Nater et al. (2009) studied peripheral blood mononuclear cells from 10 healthy men at baseline and 0, 30, and 60 min and reported time-dependent changes in immune and signaling pathways. Jurkiewicz et al. (2021) analyzed peripheral blood mononuclear cells from 12 cortisol responders at baseline and two post-TSST time points and likewise observed dynamic mRNA and microRNA responses. Schwaiger et al. (2016) examined purified monocytes in 60 adults stratified by childhood-adversity history and found stress-responsive enrichment involving cytokine, chemokine, and receptor-signaling functions. Logan et al. (2022) compared 22 women exposed to the TSST with 18 non-stress controls and identified 11 differentially expressed genes. Differences in cell source (whole blood, PBMCs, or monocytes), sample composition, control design, sampling schedule, platform, and statistical pipeline likely contribute to the limited gene-level overlap. Our repeated whole-blood sampling with contemporaneous hematology extends the temporal description.

### 4.2 Slow gene expression responses to stress

We found a set of genes that showed gradual, linear upregulation with no pronounced peak following stress (e.g., *CXCR2, QPCT, AQP9, FPR2, SULT1A1* and *PLAUR*). Many of these genes are expressed predominantly in neutrophils and are involved in processes such as chemotaxis, migration, metabolism and degranulation. Notably, the trajectories of these genes did not consistently parallel the observed neutrophil counts. This dissociation suggests that the observed transcriptional changes cannot be fully explained by shifts in blood cell composition alone and instead point toward cell-intrinsic responses.

Activation of motor neurons during acute stress has been shown to increase neutrophil counts in mice 1 to 2 h later (Poller et al., 2022). The activation of motor neurons released the *CXCR2* ligand CXCL1 which is an attractor of neutrophils. We observed an increase in expression of *CXCR2*. We also observed an increase in the number of neutrophils in our sample 60 to 90 min following stress. The linear temporal pattern of *CXCR2* expression was not concordant with the biphasic trajectories of neutrophil counts, suggesting that *CXCR2* expression is cell intrinsic and not explained by cell counts alone. Recently, Dohi et al. (2024) demonstrated that acute stress induced strong increases of CXCL1/-2 and CXCR2 in the cervical lymph nodes of male Sprague-Dawley rats which increased mRNA levels of *CXCR2*. The temporal pattern of *CXCR2* expression observed in our data showed a similar trend between baseline and post-stress timepoints. Based on these findings in rodents and our findings in humans, we speculate that IL-8 (human CXCR2 ligand) release in blood following stress leads to increased neutrophil recruitment and succeeding upregulation of *CXCR2*.

### 4.3 Biological pathways and transcription regulators

Our combined analyses of protein interactions, gene sets, pathways and transcription factors suggest that immune activity in T-cells and neutrophils play a key role in the gene expression changes that follow acute stress. Our GSEA results revealed enrichment of biological processes related to cytotoxic and secretory immune functions, including granzyme-mediated programmed cell death, immune response, killing of cells of another organism, and several granule-associated gene ontology terms (cytolytic granule, secretory granule lumen, mast cell granula, and extracellular region). Similarly, our protein-protein interaction analysis indicated enrichment for processes such as regulation of extrathymic T-cell differentiation, NK-cell-mediated immunity, cytolytic granule formulation, and cytotoxic T-cell pyroptotic processes, all pointing to enhanced cytotoxic and inflammatory activity. Further analysis of inferred transcription factor regulation using ChEA3 highlighted IL-12 family-related regulatory signatures, suggesting that stress may transiently enhance Th1-type immune activation involving cytotoxic T-cells, NK-cells and neutrophils.

IL-12 is a key proinflammatory cytokine and a potent driver of type 1 immune responses, known for activating NK-cells (Andreadou et al., 2023; Shaashua et al., 2014). Beyond its immunological role, IL-12 contributes to neuroinflammatory processes implicated in major depressive and anxiety disorders (Drevets et al., 2022). On the other hand, it can either exacerbate or alleviate inflammation in central nervous system autoimmune conditions (Andreadou et al., 2023; Sun et al., 2022). In patients with panic disorder, IL-12 and IL-1β levels were found to correlate negatively with cortisol, indicating that stress-related hormonal responses may directly inhibit proinflammatory cytokine activity (Fernández‐Serrano et al., 2022). IL-12 primarily signals through IL-12-STAT4 activation, which has been shown to mediate most physiological effects of IL-12 (Sun et al., 2015). Consistent with these findings, our transcription factor analysis identified STAT4 as one of the top regulators. STAT4 was further linked to several genes associated with cytotoxic and inflammatory functions, including *GZMA, CCL4, GZMB, CD3D, NKG7, CST7, MT2A*, and *PLAUR*, indicating coherence between inferred transcriptional regulation and known IL-12/STAT4 immune programs.

Another notable transcription factor identified was *TBX21* (T-bet), a key regulator of lymphocyte differentiation and function that plays a pivotal role in programming CD8^+^-cells and NK-cells (Intlekofer et al., 2005). T-bet may also mediate behavioral responses to stress. In mice, deficiency of T-bet markedly reduced anxiety- and depression-like behaviors following acute stress exposure, accompanied by lower serum levels of the proinflammatory cytokines IL-6 and TNF-⍺ (Kim et al., 2011). This supports a potential genetic link, *TBX21* polymorphisms have also been associated with major depressive disorder (Wong et al., 2008).

### 4.4 Clinical implications for anxiety and depression

Cytokines have long been proposed to mediate the connection between stress, anxiety and depression (Loftis et al., 2010). The IL-12 family signaling pathway may be particularly important in this context due to its interaction with the HPA-axis (Shaashua et al., 2014). Elevated plasma IL-12 levels have been reported in individuals with major depressive disorder (Köhler et al., 2017), and treatment with antidepressants can reduce IL-12 concentrations in patients with depression (Xu et al., 2023) and anxiety (Hou et al., 2019), although this reduction has not been observed in all studies (Liu et al., 2020). Interestingly, drugs used to treat psoriasis that act on the IL-12 cytokine family, such as ustekinumab and guselkumab, have also been found to alleviate symptoms of anxiety and depression (Kim et al., 2011; Gordon et al., 2018; Langley et al., 2010). While these effects may partly reflect relief from psoriasis-related distress, they could also result from reduced IL-12 activity. Collectively, such findings suggest that pharmacological inhibition of the IL-12 pathway might hold potential as an adjunct therapy for depression and anxiety disorders (Drevets et al., 2022; Sarmin et al., 2024).

Our findings further identify specific genes that may help explain how stress relates to anxiety and depression, with particular attention to *PLAUR*. In a knock-out model, it has been shown that mice lacking uPAR (the protein encoded by *PLAUR*) display increased anxiety-like behaviors as compared to their wild type counterpart (Powell et al., 2003). In humans, a recent UK Biobank study linked higher plasma *PLAUR* protein levels to an elevated risk of developing anxiety or depression over a 14-year period, and identifier *PLAUR* as a key inflammatory mediator in their comorbidity (Ding et al., 2025). Elevated *PLAUR* levels have also been associated with current depression and PTSD in the same cohort (Daskalakis et al., 2024; Kang et al., 2025). In gene expression studies, *PLAUR* was among the core enrichment genes distinguishing men with generalized anxiety disorder from controls (Wingo and Gibson, 2015). Consistently, increased *PLAUR* RNA expression has been observed in postmortem brains of individuals with major depressive disorder and PTSD (Daskalakis et al., 2024), as well as across multiple neural and endothelial cell types in the dorsolateral prefrontal cortex (Hwang et al., 2025). Our results link these findings to the slower component of the stress response, which could suggest an involvement of neutrophils in anxiety and depression, as well as more general stress related processes occurring in many different cell types, including brain cells.

Another notable gene identified in our analyses was *GRN*, which encodes progranulin (PGRN), which has been implicated in multiple studies of anxiety. In mice, sex-specific differences in anxiety behaviors have been observed in wildtype, but not in Pgrn knockout animals, suggesting that PGRN contributes to the generation of sex differences in anxiety-related behaviors (Chiba et al., 2009). Progranulin also interacts with TNF-⍺ receptors, and an imbalance between PGRN and TNF-⍺ has been shown to induce memory impairments and anxiety-like behaviors in sleep-deprived mice (Zhang et al., 2017). Furthermore, treatment with either exogenous PGRN or a TNF-⍺ inhibitor reversed these effects, while PGRN also suppressed dendritic spine loss and restored neurogenesis in a specific part of the hippocampus in these mice, indicating a neuroprotective role (Zhang et al., 2017). More recently, intracerebroventricular administration of PGRN following cerebral ischemia reduced anxiety-like behavior, and improved spatial learning, memory and hippocampal neurogenesis, suggesting its potential as a therapeutic candidate for mood and cognitive impairments after ischemic stroke (Sun et al., 2022). Taken together, these findings implicate *GRN*/PGRN as a key factor in the interaction between stress, inflammation, and anxiety regulation.

We also observed gradual increased expression of glutamine synthetase (*GLUL*), which plays a key role in neurotransmission by recycling the excitatory neurotransmitter glutamate, and has been implicated in both stress and depression, as well as neurological disorders such as schizophrenia and epilepsy (Mundorf et al., 2019; Rose et al., 2013). During acute stress, glutamate release increases, followed by enhanced recycling through glutamine synthetase, a process tightly regulated by glucocorticoids (Popoli et al., 2012). Postmortem studies have shown decreased expression of *GLUL* in glial cells in individuals with depression, suggesting disruptions in glutamate metabolism may underly depressive pathology (Choudary et al., 2005). In male mice, lithium treatment showed sex-specific protection against stress-induced neurobiological changes, seen through a change in glutamine synthetase activity levels via a gene promoter reporter not observed in female mice (Mundorf et al., 2019). Additionally, injection-induced stress alone increased promoter activity of *GLUL*, highlighting its sensitivity to acute stress exposure (Mundorf et al., 2019). Altogether, these findings underscore the therapeutic potential of targeting the glutamate-glutamine cycle, as pharmacological modulation of glutamate synapses may offer alternatives conventional monoaminergic treatments for mood disorders (Popoli et al., 2012; Chrobak and Siwek, 2024).

*FPR2* was another gene that increased slowly after stress. *FPR2* encodes the formyl peptide receptor 2 and is expressed by monocytes and neutrophils, where its expression is enriched during degranulation (Pontén et al., 2008). The FPR2 protein can recognize a wide variety of ligands, through which it can act as either a pro- or anti-inflammatory modulator and participate in the resolution of inflammation in the central nervous system (Tylek et al., 2021). In mice, *FPR* signaling has been shown to strongly impact anxiety-like behaviors (Gallo et al., 2014; Zheng et al., 2025). For example, one recent mouse study demonstrated that treatment with an *FPR* antagonist (synthetic peptide WRW4) mitigated anxiety- and depression-like behaviors in a social isolation mouse model (Zheng et al., 2025).

Several of the genes that changed expression rapidly after stress have also been linked to anxiety and depression. For example, downregulated *GZMB* expression has been observed in individuals with current depression as compared to those in remission (Jansen et al., 2016), while blood levels of the cytokine CCL4 has been found to be elevated in individuals with anxiety, depressive disorders, and *PTSD* (Camacho-Arroyo et al., 2021; de Koning et al., 2022; Ogłodek and Just, 2018). This suggests that both genes that change expression shortly after stress, and genes that show more delayed expression changes, have been found to be associated with stress-related disorders.

## 5 Limitations

Several limitations constrain interpretation. Importantly, as the study did not include a time-matched non-stress control condition, it is not possible to ascertain that observed transcriptional changes were uniquely attributed to the TSST or represent natural fluctuations over time. Furthermore, the TSST is known to elicit sex specific effects (Liu et al., 2017; Kelly et al., 2008). The sample size in this study, however, was too small to analyse sex differences with adequate power. Finally, for practical reasons and to limit the burden on participants, sampling was restricted to 90 min post stress. To investigate the full duration of observed changes, sampling at even later time points is needed.

## 6 Conclusion

In summary, our results show that acute social stress induces both rapid and delayed transcriptional changes across immune pathways. The early response reflects activation of cytotoxic lymphocyte programs, while the later response involves neutrophil-associated transcriptional regulation, both implicating IL-12 family signaling associated regulatory programs. These findings reveal a coordinated temporal structure of immune gene activation following psychosocial stress and identify molecular targets such as STAT4 and *PLAUR* that may bridge peripheral immune activation with neurobiological processes underlying anxiety and depression.

## CRediT authorship contribution statement

**Hampus Grönvall:** Conceptualization, Data curation, Formal analysis, Investigation, Methodology, Project administration, Visualization, Writing – original draft, Writing – review & editing. **Sylvia Abdelhalim:** Investigation, Project administration, Writing – review & editing. **Fara Tabrizi:** Conceptualization, Investigation, Methodology, Project administration, Writing – review & editing. **Sini Ezer:** Writing – review & editing. **Gamze Yazgeldi Gunaydin:** Methodology, Visualization, Writing – review & editing. **Erik Arner:** Conceptualization, Writing – review & editing. **Jörgen Rosén:** Conceptualization, Investigation, Writing – review & editing. **Shintaro Katayama:** Methodology, Writing – review & editing. **Juha Kere:** Conceptualization, Writing – review & editing. **Fredrik Åhs:** Conceptualization, Funding acquisition, Investigation, Methodology, Project administration, Resources, Supervision, Writing – original draft, Writing – review & editing. **Lea Mikkola:** Conceptualization, Formal analysis, Methodology, Writing – original draft, Writing – review & editing.

## Ethics declaration

Written informed consent to take part in the study and to publish the article has been obtained from all participants or their legal representatives. The privacy rights of participants have been observed.

This study included organ or tissue donors. This study includes human biological material and consent was obtained by donors, or their next of kin or legal representatives, for use in this study and for publication of the article. The samples used in this research were not sourced from executed prisoners or prisoners of conscience.

This study was performed in compliance with relevant laws, regulatory frameworks and guidelines where the research took place. This study was approved by the Swedish Ethical Review Authority. (Approval No. Dnr. 2020-00885)

## Declaration of competing interest

The authors declare no conflict of interest.
